# Longitudinal Epidemiology and Variant Dynamics of SARS-CoV-2 in Coastal Kenya (2020–2025): Clinical Features and Wave Patterns

**DOI:** 10.1093/ofid/ofag084

**Published:** 2026-02-18

**Authors:** Arnold W Lambisia, Joyce Nyiro, George Githinji, Esther N Katama, Edidah Moraa, John M Mwita, Martin Mutunga, Grace Maina, Philip Bejon, My V T Phan, Matthew Cotten, Simon Dellicour, L Isabella Ochola-Oyier, Charles Sande, Edward C Holmes, James Nyagwange, Charles N Agoti

**Affiliations:** Epidemiology and Demography Department, Kenya Medical Research Institute–Wellcome Trust Research Programme, Kilifi, Kenya; Epidemiology and Demography Department, Kenya Medical Research Institute–Wellcome Trust Research Programme, Kilifi, Kenya; Epidemiology and Demography Department, Kenya Medical Research Institute–Wellcome Trust Research Programme, Kilifi, Kenya; Department of Biochemistry and Biotechnology, School of Public Health, Pwani University, Kilifi, Kenya; Epidemiology and Demography Department, Kenya Medical Research Institute–Wellcome Trust Research Programme, Kilifi, Kenya; Epidemiology and Demography Department, Kenya Medical Research Institute–Wellcome Trust Research Programme, Kilifi, Kenya; Epidemiology and Demography Department, Kenya Medical Research Institute–Wellcome Trust Research Programme, Kilifi, Kenya; Epidemiology and Demography Department, Kenya Medical Research Institute–Wellcome Trust Research Programme, Kilifi, Kenya; Epidemiology and Demography Department, Kenya Medical Research Institute–Wellcome Trust Research Programme, Kilifi, Kenya; Epidemiology and Demography Department, Kenya Medical Research Institute–Wellcome Trust Research Programme, Kilifi, Kenya; Nuffield Department of Medicine, University of Oxford, Oxford, UK; College of Health Solutions, Arizona State University, Phoenix, Arizona, USA; College of Health Solutions, Arizona State University, Phoenix, Arizona, USA; Complex Adaptive System Initiative, Arizona State University, Scottsdale, Arizona, USA; Spatial Epidemiology Lab, Université Libre de Bruxelles, Bruxelles, Belgium; Department of Microbiology, Immunology and Transplantation, Rega Institute and Laboratory for Clinical and Epidemiological Virology, KU Leuven, University of Leuven, Leuven, Belgium; Interuniversity Institute of Bioinformatics in Brussels, Université Libre de Bruxelles, Vrije Universiteit Brussel, Brussels, Belgium; Epidemiology and Demography Department, Kenya Medical Research Institute–Wellcome Trust Research Programme, Kilifi, Kenya; Nuffield Department of Medicine, University of Oxford, Oxford, UK; Epidemiology and Demography Department, Kenya Medical Research Institute–Wellcome Trust Research Programme, Kilifi, Kenya; Nuffield Department of Medicine, University of Oxford, Oxford, UK; School of Medical Sciences, University of Sydney, Camperdown NSW 2050, Sydney, Australia; Epidemiology and Demography Department, Kenya Medical Research Institute–Wellcome Trust Research Programme, Kilifi, Kenya; Epidemiology and Demography Department, Kenya Medical Research Institute–Wellcome Trust Research Programme, Kilifi, Kenya; Department of Biochemistry and Biotechnology, School of Public Health, Pwani University, Kilifi, Kenya

**Keywords:** coastal Kenya, COVID-19, SARS-CoV-2, symptoms, waves

## Abstract

**Background:**

SARS-CoV-2 is a major cause of outpatient-attended acute respiratory infections (ARIs). Data from Africa are limited on SARS-CoV-2 infection, variants, symptom profile, and longitudinal trends for outpatient presentation.

**Methods:**

Starting December 2020, we established ARI surveillance at 5 outpatient clinics in coastal Kenya, recruiting ∼15 participants (any age) per week per clinic for SARS-CoV-2 testing and genome analysis. Participants provided respiratory samples, demographic details, and vaccination and symptom data. We compared SARS-CoV-2 clinical and molecular epidemiology before and during Omicron waves using multivariate logistic regression.

**Results:**

By February 2025, we had recruited 14 562 ARI cases, with 1053 (7.2%) testing positive for SARS-CoV-2. The median age of cases was 25 years (IQR, 15–41) and 65.0% were female. Nine infection waves were recorded, with positivity ranging 8.2% to 25.6%. Interwave intervals increased from ≤3 months in 2021 to ≥6 months in 2024. Sixty-eight PANGO lineages were identified from 782 (74.2%) sequenced cases, with 4 predominating local waves (AY.116, BQ.1.8, FY.4.1, LF.7.3.2), which were rare globally (<0.5%) during their detection period. Overall, common symptoms among positive cases were cough (91.5%), nasal discharge (76.7%), and fever (53.1%). Loss of sense of smell was strongly predictive of COVID-19 in the pre–Omicron era, but body malaise, sore throat, joint pain, and nasal discharge were predictive during the Omicron period.

**Conclusions:**

SARS-CoV-2 increasingly shows seasonal annual patterns in coastal Kenya, with its clinical features resembling established endemic respiratory viruses. Its case burden is most pronounced in young adults. Locally dominant genetic variants may differ from those globally.

## Teaser

Clinical and genomic analysis of SARS-CoV-2 outpatient infections in coastal Kenya (2020–2025) revealed widening interwave intervals, repeated predominance of locally distinct PANGO lineages, and shifting of predictive symptoms following Omicron emergence, thereby underscoring the importance of continued local SARS-CoV-2/respiratory virus surveillance.

An understanding of the long-term patterns of severe acute respiratory syndrome coronavirus 2 (SARS-CoV-2) infection waves is only beginning to emerge [1, 2]. The virus has sustained recurrent community outbreaks 5 years into its emergence in human populations, adding further strain on health care systems, especially in low- and middle-income countries (LMICs) [3, 4].

Globally, successive waves of SARS-CoV-2 infections in communities have been typically dominated by a distinct variant [5]. However, only a limited number of locations have sustained SARS-CoV-2 surveillance beyond the acute pandemic period (2020–2022) to provide insights into emerging variants, wave periodicity, and spatial-temporal lineage dynamics [6]. In the postpandemic period, there is a general paucity of data from LMICs on infection frequency (prevalence), demographics (eg, age patterns), circulating genetic variants (LMICs contribute ∼0.1% of postpandemic data to the Global Initiative on Sharing All Influenza Data [GISAID]) [6], as well as clinical presentation of the infection. This has resulted in a limited understanding of ongoing COVID-19 disease burden and clinical presentation [7].

Since 2020, COVID-19 clinical presentation has continuously evolved, with changes in circulating variants, existing countermeasures, and host immunity [8–10]. Earlier in the pandemic, the losses of smell and taste were telling of SARS-CoV-2 infection [8]. However, with the arrival of Omicron came a transition to more typical upper respiratory, systemic, and neurologic symptoms in the absence of impaired senses of smell and taste [9, 11]. Thus, linking clinical and genomic surveillance for COVID-19 cases offers an opportunity for optimal clinical diagnosis and patient care [3, 11].

Since December 2020, we have maintained acute respiratory illness (ARI) surveillance across 5 selected outpatient health facilities in Kilifi, coastal Kenya [12]. Our previous analysis showed cocirculation of multiple PANGO lineages during new waves [13], most of which were newly introduced but did also include some that appear to have evolved locally and then spread globally [3, 14, 15]. Leveraging this platform, we elucidate the longer-term molecular-epidemiologic trends of SARS-CoV-2 in the region, including wave timings, and assess the changes in clinical presentation over successive waves to inform future control strategies [12]. The lineage composition of the most recent local wave (wave 10) is also described for the first time.

## METHODS

### Study Site and Selected Facilities

**Figure 1.**
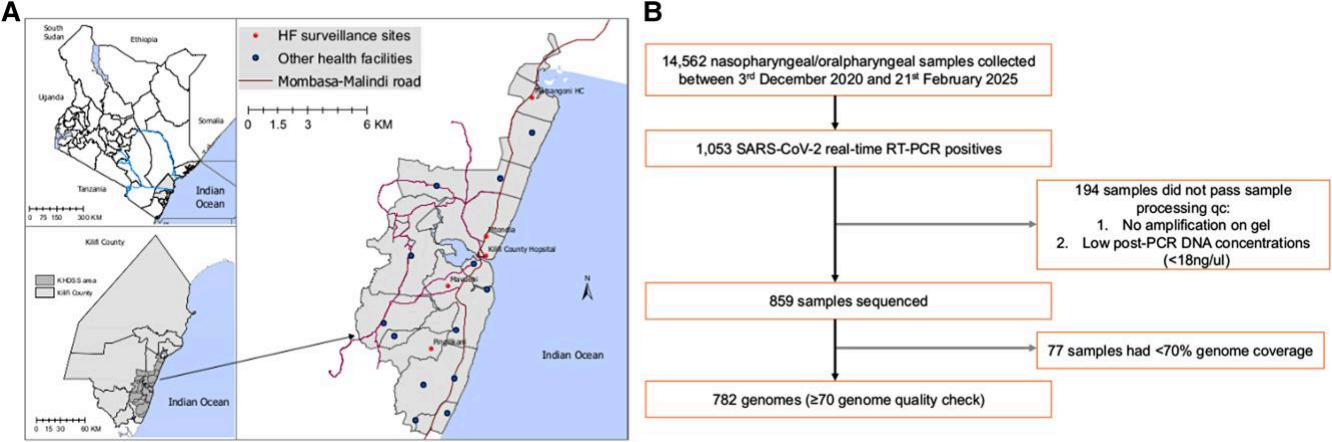
Study area map and sample flowchart. *A*, Map of the study area shows Kenya, Kilifi County, and the Kilifi Health and Demographic Surveillance System (KHDSS) area within Kilifi, Kenya, where the study was undertaken. The location of the 5 health facilities (HFs) that conducted outpatient surveillance is indicated. *B*, Flow diagram of sample collection and screening from December 2020 to February 2025. qc, quality control; RT-PCR, reverse transcription polymerase chain reaction.

The study was conducted within the Kilifi Health and Demographic Surveillance System area, coastal Kenya, which has approximately 300 000 residents, leveraging the outpatient ARI surveillance platform (Figure 1) [16]. Here, we aimed to recruit 15 participants per week from each of the 5 participating outpatient health facilities: Kilifi County Referral Hospital (the outpatient department draws patients from all over Kilifi County), Matsangoni, Mtondia, Mavueni, and Pingilikani. The recruitment is on a first-come, first-enrolled basis. Thus, approximately 75 participants are recruited across the 5 health facilities per week. The analysis presented here is between December 2020 and February 2025. Participants were recruited if they presented with fever (history or measured, >38.0 °C) and ≥1 ARI symptoms (Supplementary material) and provided written informed consent. Newborns (<1 month), individuals who had ARI symptoms for >28 days, and those who declined consent were excluded.

### Laboratory Procedures

Nasopharyngeal and/or oropharyngeal samples were received at Kenya Medical Research Institute–Wellcome Research Programme within 24 hours following collection. Total nucleic acids were extracted and screened for SARS-CoV-2 as previously described [17]. Briefly, viral RNA was extracted by either an automated Qiacube HT with a RNeasy extraction kit (Qiagen) or manually with the QIAamp Viral RNA Mini Kit (Qiagen), and screening was done with multiple commercial real-time reverse transcription polymerase chain reaction kits depending on availability during the study period [18, 19]. Samples were defined as positive based on the assay-specific polymerase chain reaction cycle threshold cutoffs. Whole genome amplification was attempted on all positive samples (cycle threshold <35.0). Those that were amplified successfully and passed quality control checks (Supplementary material) were sequenced on either a GridION (Oxford Nanopore Technologies) or MiSeq (Illumina) sequencing platform, depending on the kits that were available in the laboratory at the time [17]. Further details are provided in the Supplementary material.

### Genomic Data Analysis

Raw sequence read data were assembled to generate consensus genomes through the ARTIC bioinformatic pipeline for Oxford Nanopore Technologies reads (https://artic.readthedocs.io/en/latest/) or an in-house bioinformatic pipeline for Illumina paired-end reads [3] as described previously [3] (detailed workflows in the Supplementary material). By using NextClade version 3.10.2 and the data set “nextstrain/sars-cov-2/wuhan-hu-1” version 2025-06-09-15-42-38Z in the command line, sequence quality was checked, and PANGO lineages and clades were assigned to the consensus genomes [20]. Sequences with genome coverage <70% were excluded from downstream analysis [17].

Using GISAID data to compare the similarity between local and global PANGO lineage trends, we determined if the dominant lineages observed during a local wave in Kilifi were from the top 5 lineages circulating globally during the corresponding periods. All genomic data (17.1 million sequences) and associated metadata deposited up to 28 February 2025 were downloaded from GISAID [6]. The data were binned by dates of locally observed wave periods and subsampled to achieve 100 sequences per wave, month of collection, and country, generating a total of 6 792 560 sequences. These data were then stratified by PANGO lineage to identify the dominant lineages globally across each wave interval relative to the coastal Kenya patterns.

### Statistical Analyses

A multivariate analysis was conducted in which 4 outcomes were evaluated: individual SARS-CoV-2 infection status, SARS-CoV-2 waves of infection, SARS-CoV-2 variant distribution patterns, and symptom profiles. The outcomes were analyzed over the study period and compared across 2 major periods (pre-Omicron and Omicron epidemic waves). The pre-Omicron period spanned December 2020 to October 2021, while the Omicron period began when the first Omicron cases were observed (BA.1 lineage; November 2021) and extended to February 2025. Weekly trends of SARS-CoV-2 cases and PANGO lineages were summarized across the study period. The positivity rate was estimated within each wave, defined as a period beginning when the weekly positivity rate increased by ≥10% for 2 consecutive weeks and ending when the positivity rate declined for 2 weeks and returned to within 5% of the wave’s starting level [21, 22].

To compare demographic characteristics (age group, sex, and facility) between individuals who were SARS-CoV-2 positive and negative, a χ^2^ or Fisher exact test was used as appropriate. Multivariable logistic regression models were constructed to assess associations between individual symptoms and SARS-CoV-2 infections, adjusting for age, sex, and study period. The model equation was as follows:


$$\log\;\mathrm{it}(P)=\beta_{0}+\beta_{1}X_{1}+\beta_{2}X_{2}+\beta_{3}X_{3}+\beta_{4}X_{4},$$

where

β₀ = intercept

β₁, β₂, β₃, β₄ = regression coefficients

*P* represents the probability of SARS-CoV-2 infection status (0 = negative, 1 = positive)

*X*₁ = individual symptom (no, yes)

*X*₂ = age (continuous variable, years)

*X*₃ = sex (female, male)

*X*₄ = study period (pre-Omicron and Omicron)

Separate models were built for the pre-Omicron and Omicron periods, in addition to an overall model spanning the entire study. Age, sex, and period (pre-Omicron and Omicron) were adjusted for in the models. Results are reported as adjusted odds ratio and 95% CI, with *P* < .05 considered statistically significant. Fifteen cases with missing symptom data were removed before performing multivariable analysis. Multicollinearity was assessed by variance inflation factor. The area under the curve was calculated as a summary measure of model discrimination.

## RESULTS

Between 3 December 2020 and 21 February 2025, a total of 14 562 nasopharyngeal and/or oropharyngeal samples were collected from patients presenting with ARI at 5 outpatient health facilities in coastal Kenya (Table 1). Of these, 1053 (7.2%) samples were positive for SARS-CoV-2. There was a significant difference in the distribution of SARS-CoV-2 cases across study years (*P* < .001) and facilities (*P* = .002). Among the 1053 positives, 859 (81.5%) passed sample-processing quality control checks and were sequenced; 77 sequenced samples with genome coverage <70% coverage were dropped from downstream lineage analyses (Figure 1).

**Table 1.** Baseline Characteristics of Positive and Negative SARS-CoV-2 Cases Presenting With ARI in 5 Outpatient Facilities in the KHDSS Between December 2020 and February 2025

	Positive (n = 1053)	Negative (n = 13 509)	Total (n = 14 562)	*P* Value
Sex: female	684 (65.0)	8336 (61.7)	9020 (61.9)	.037
Age, y, median (IQR)	25 (15–41)	17 (7–33)	18 (7–34)	
Age group, y				<.001
0–4	106 (10.1)	2553 (18.9)	2659 (18.3)	
5–9	66 (6.3)	1643 (12.2)	1709 (11.7)	
10–19	210 (19.9)	3338 (24.7)	3548 (24.4)	
20–39	372 (35.3)	3170 (23.5)	3542 (24.3)	
40–64	230 (21.8)	2038 (15.1)	2268 (15.6)	
≥65	69 (6.6)	767 (5.7)	836 (5.7)	
Year of collection^a^				<.001
2020		4 (0.0)	4 (0.0)	
2021	351 (33.3)	2501 (18.5)	2852 (19.6)	
2022	353 (33.5)	3275 (24.2)	3628 (24.9)	
2023	181 (17.2)	3566 (26.4)	3747 (25.7)	
2024	159 (15.1)	3624 (26.8)	3783 (26.0)	
2025	9 (0.9)	539 (4.0)	548 (3.8)	
Health facility				.002
Kilifi County Hospital	247 (23.5)	3074 (22.7)	3321 (22.8)	
Matsangoni	195 (18.5)	2569 (19.0)	2764 (19.0)	
Mavueni	179 (17.0)	2686 (19.9)	2865 (19.7)	
Mtondia	241 (22.9)	2472 (18.3)	2713 (18.6)	
Pingilikani	191 (18.1)	2708 (20.0)	2899 (19.9)	
Vaccination status^b^				…
Yes	141 (13.4)	1552 (11.5)	1693 (11.6)	
No	587 (55.7)	9803 (72.6)	10 390 (71.4)	
No data	325 (30.9)	2154 (15.9)	2479 (17.0)	

Data are presented as No. (%) unless indicated otherwise.

Abbreviations: ARI, Acute Respiratory Infection; KHDSS, Kilifi Health and Demographic Surveillance System.

^a^Data from 2020 were collected over just a 1-month period, while data from 2025 were collected over a 2-month period (January and February).

^b^17% of the participants have missing data, and comparison was not done between positive and negative cases of SARS-CoV-2.

Positive and negative cases of SARS-CoV-2 differed in their sex distribution (female: 65.0% and 61.7%, respectively; *P* = .037) and age (median, 25 vs 17 years; *P* < .001) as shown in Table 1. Three age groups also had greater representation in positive cases: 20 to 39 years (35.3% vs 23.5%), 40 to 64 (21.8% vs 15.1%), and ≥65 (6.6% vs 5.7%). Only 11.6% of the recruited participants reported receiving COVID-19 vaccination with AstraZeneca, Pfizer, Moderna, and Johnson & Johnson.

**Figure 2.**
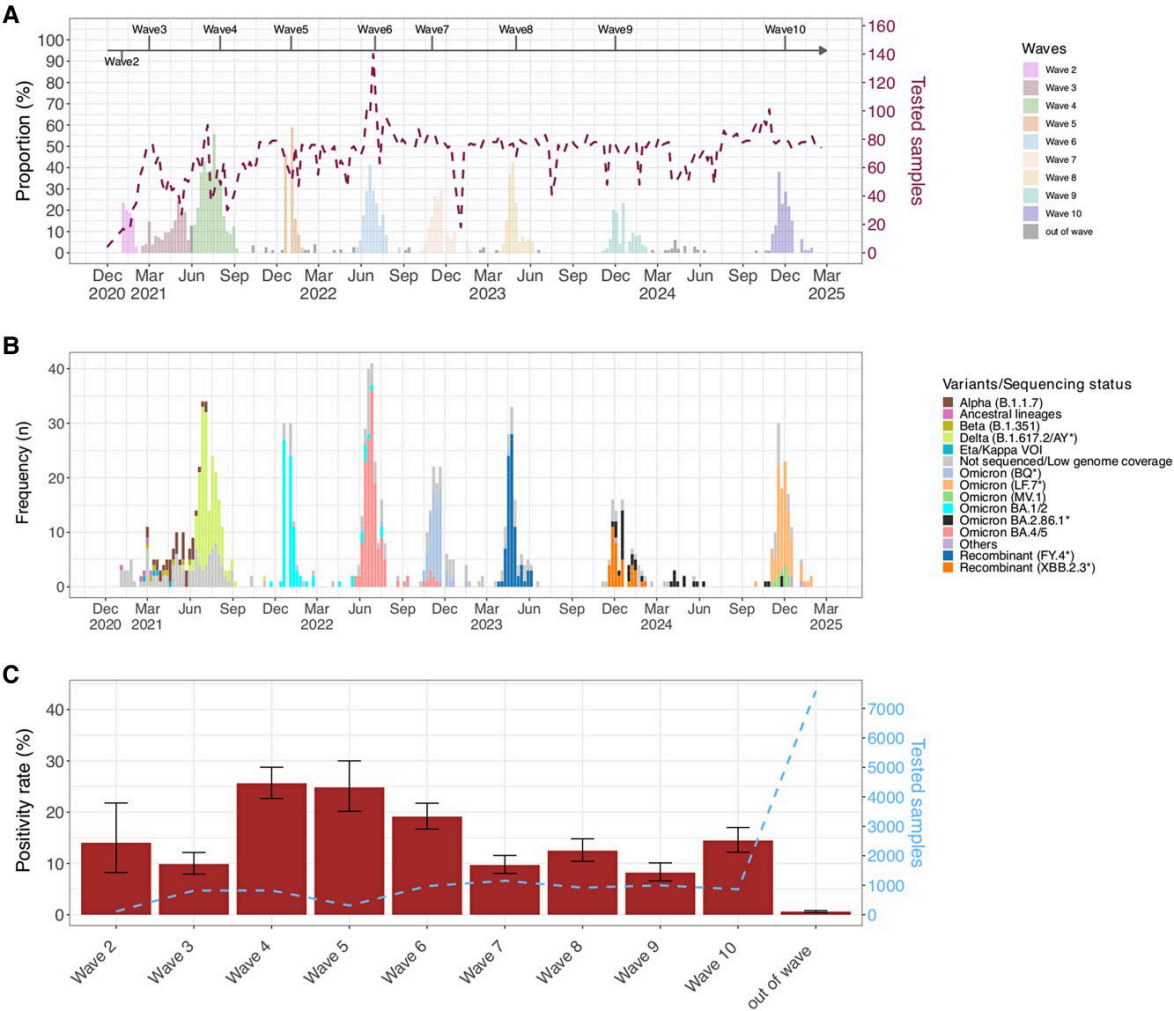
Temporal trends of SARS-CoV-2 cases, wave positivity rates, and PANGO lineage. *A*, Weekly temporal trends of SARS-CoV-2 cases from December 2020 to February 2025 in 5 outpatient facilities with the Kilifi Health and Demographic Surveillance System in Kilifi, Kenya. The dashed brown line shows the number of samples tested per week (secondary y-axis), and the bars showing the weekly proportion of tested samples that were positive are colored by waves. *B*, Weekly temporal trends of SARS-CoV-2 variants identified during the study period. *C*, Positivity rate of SARS-CoV-2 across waves and out of wave. The dashed blue line shows the number of samples tested per wave (secondary y-axis). Error bars indicate 95% CI.

The first SARS-CoV-2 case in Kenya was reported on 12 March 2020. Until 21 February 2025, 10 waves of infection have been observed in Kilifi, Kenya, although the first epidemic wave recorded few infections and exhibited limited spread [13, 23, 24]. Here, we analyzed data collected between 3 December 2020 and 21 February 2025, spanning 9 waves of infection (Figure 2*A*). During wave periods, the SARS-CoV-2 positivity rate ranged from 8.2% (95% CI, 6.5%–10.1%) to 25.6% (95% CI, 22.6%–28.7%). In contrast, in nonwave periods, the prevalence was 0.6% (95% CI, .4%–.8%; Figure 2*C*). In 2021, 4 waves of infection were observed. These were predominated by the variants B.1.530, B.1.1.7 (Alpha), AY.116 (Delta), and BA.1.1 (Omicron), with their intervals ranging from 2 weeks to 3 months. In 2022 and 2023, the interval between consecutive waves gradually increased to approximately 5 months, and by 2024 to 2025, waves were observed 9 months apart. The average duration of waves was 2.8 months (range, 1.2–3.7 months).

### Distribution of Lineages Across Waves

**Figure 3.**
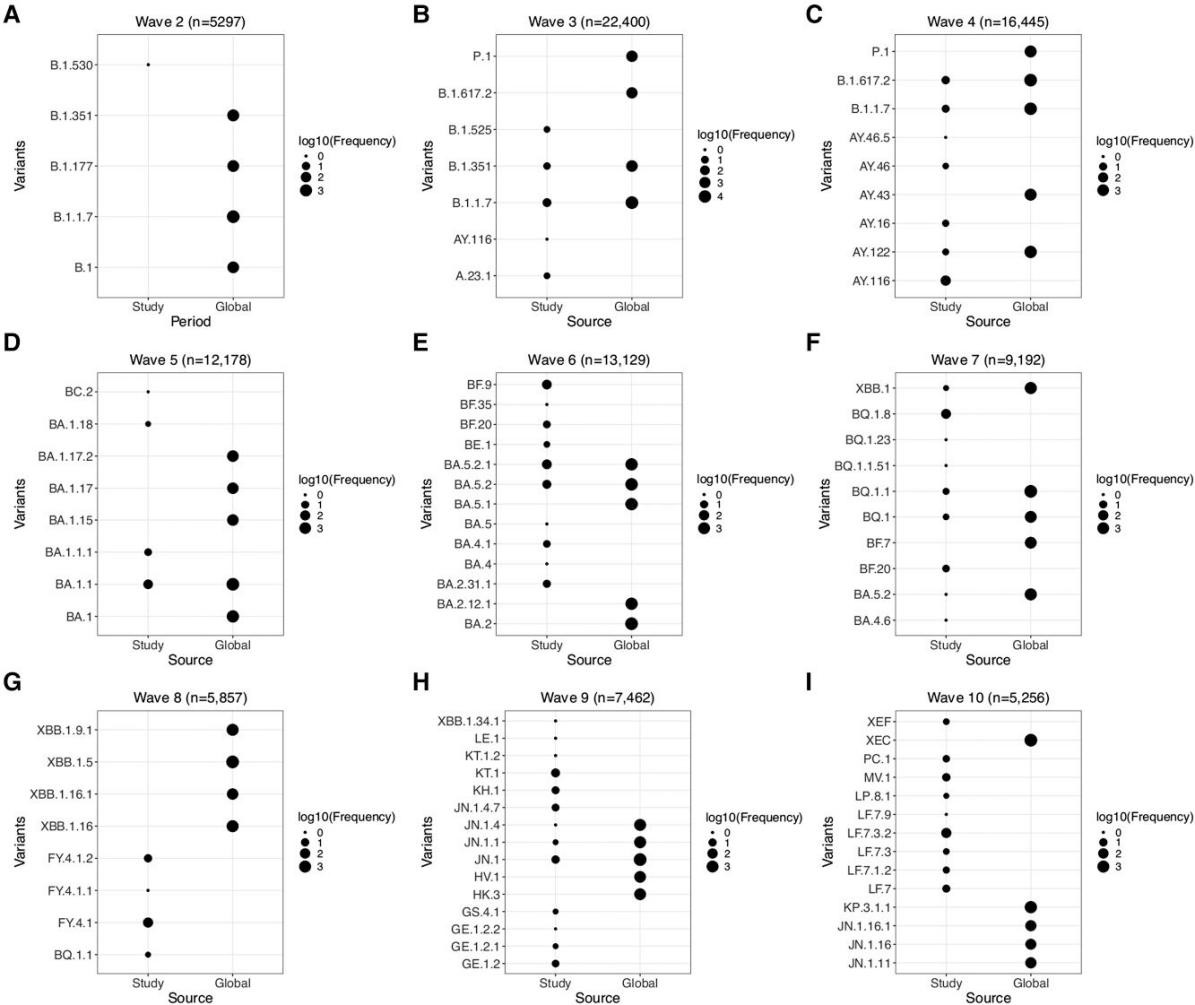
Temporal patterns of PANGO lineages across waves in Kilifi, Kenya, and globally between December 2021 and February 2025. The PANGO lineages are grouped by wave from waves 2 to 10 (*A* to *I*) and plotted on the y-axis with source of sequences on the x-axis.

Between 8 January 2021 and 28 January 2025, 68 PANGO lineages were identified from the 782 sequenced cases (Figure 2*B*, Supplementary Figure 1). A median of 7 (IQR, 5–9) PANGO lineages were detected in each wave with 1 or 2 predominant lineages (Figure 3). In waves 3 and 5, similar lineages were dominant locally and globally: Alpha (B.1.1.7; local = 64%, global = 51%) and Omicron (BA.1.1, 82% vs 30.4%). During wave 6, there was joint dominance of 2 lineages locally, BA.5.2.1 (35%) and BF.9 (an alias of BA.5.2.1.9; 32.4%), while at a global level, BA.5.2.1 (7.3%) was among the top circulating lineages whereas BF.9 was less common (0.01%). Similarly, during wave 9, there was joint dominance of XBB.2.3-derived lineages (KT.1, 31.7%; KH.1, 12.6%; GE.1.2, 9.5%) and JN.1-like lineages (JN.1, 17.4%; JN.1.4.7, 11.1%), composing 82.3% of the sequenced genomes, whereas globally only JN.1 was the top lineage detected (21.6%). During this wave, the XBB.2.3-like lineages constituted <0.01% of the global lineages. During waves 4, 7, 8, and 10, B.1.617.2 (14.1%), BQ.1.1 (alias BA.5.3.1.1.1.1.1.1, 13.5%), XBB.1.5 (16.9%), and XEC (37.5%) were the top lineages detected globally, while AY.116 (77.1%), BQ.1.8 (76.2%), FY.4.1 (alias XBB.1.22.1.4.1, 85.1%), and LF.7.3-like (61.4%) were dominant locally (Figure 3). Notably, the dominant local lineages during these 4 waves, AY.116, BQ.1.8, FY.4.1, and LF.7.3-like, represented <0.5% of the globally reported sequences during the same period.

### Clinical Presentation of SARS-CoV-2–Positive Cases

During the study period, cough was the most prevalent symptom among individuals seeking outpatient care who were SARS-CoV-2 positive (n = 964, 91.5%), followed by nasal discharge (n = 808, 76.7%), fever (n = 559, 53.1%), joint pain (n = 337, 32.0%), body malaise (n = 332, 31.5%), and sore throat (n = 312, 29.6%; Supplementary Figure 2). The most reported symptoms were consistent across the pre-Omicron and Omicron periods (Supplementary Figure 2A). Individuals reported a median 3 symptoms (range, 1–10) during a clinic visit (Supplementary Figure 2B). The proportion of individuals presenting each symptom by age group was calculated across age groups ≥5 years. We did not include the <5-year-olds as some symptoms (eg, headache) are difficult to capture in them. Cough, chest pain, crackles, and difficulty breathing were more prevalent in individuals ≥65 years old (Supplementary Figure 2C).

**Figure 4.**
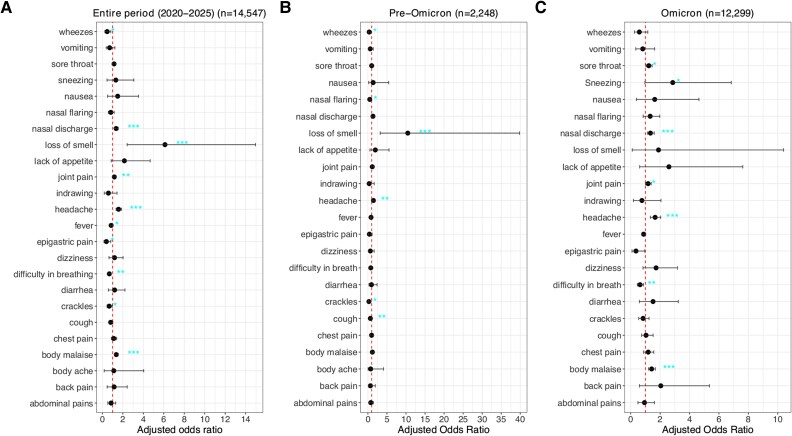
Adjusted odds ratios and 95% CIs show the association between SARS-CoV-2 infection status and individual symptoms (*A*) across the entire study period, (*B*) during the pre-Omicron period (December 2020–October 2021), and (*C*) during the Omicron period (November 2021–February 2025). *P < .05. **P < .01. ***P < .001.

The multivariable logistic regression model showed that headache, body malaise, loss of smell, nasal discharge, and joint pain were strongly associated with SARS-CoV-2 infection (Figure 4*A*, Supplementary Table 1). In contrast, fever, crackles, wheezes, epigastric pain, and difficulty breathing were negatively associated with a SARS-CoV-2 infection. No multicollinearity was observed among symptoms (all variance inflation factors <2). The area under the curve was 0.63, indicating modest discriminatory ability for the model.

When stratified by period, headache and loss of smell were significantly associated with a SARS-CoV-2 infection during the pre-Omicron period (Figure 4*B*, Supplementary Table 1). During the Omicron period, headache, body malaise, nasal discharge, sore throat, and joint pain were highly associated with SARS-CoV-2 infection (Figure 4*C*, Supplementary Table 1).

The Omicron lineages categorized into 4 groups (Supplementary Table 2)—Omicron-BA.1/2, Omicron-BA.4/5, Omicron-XBB (a recombinant variant), and Omicron-BA.2.86.1 (also known as JN.1)—showed a significant difference in distribution by age group. When compared with other Omicron variants, XBB was more prevalent among children <5 years of age and adults >65 years. The occurrence of the symptoms of body malaise, sore throat, headache, chest pain, fever, cough, nasal discharge, nasal flaring, dizziness, and joint pain differed significantly across Omicron variants (Supplementary Table 3).

As compared with Omicron-BA.2.86.1, Omicron-BA.1/2 was strongly associated with joint pain, headache, body malaise, sore throat, nasal discharge, and nasal flaring (Supplementary Table 4). Omicron BA.4/5 was strongly associated with body malaise, sore throat, chest pain, and nasal discharge. XBB-like variants were strongly associated with sore throat and cough.

## DISCUSSION

We describe the clinical and molecular epidemiology of COVID-19 over several waves of infection in coastal Kenya, December 2020 to February 2025. Such a longitudinal view of COVID-19 molecular epidemiology, lineage dynamics, and symptom profiles comparing the pre-Omicron and Omicron periods is rare in African settings [25]. Across 9 waves of infections, within-wave test positivity ranged 8% to 26% among individuals presenting with ARI in outpatient clinics. Detailed genomic analysis has been presented elsewhere for each wave but wave 10 [3, 13, 15, 26]. Here we have combined data from waves 2 to 10 to examine the longitudinal trends. We compare pre-Omicron and Omicron periods, lineage predominance at local and global scales, and clinical symptoms across multiple waves.

We reveal that patients with SARS-CoV-2 who were seeking ARI outpatient care in Kilifi, coastal Kenya, were mostly adolescents and young adults [27]. Notably, ∼67% of the population in Kilifi is aged ≤30 years as of 2023 (https://www.citypopulation.de/en/kenya/admin/coast/03__kilifi/). The highest SARS-CoV-2 positivity rate was observed during Delta (wave 4, 25.6%) and Omicron-BA.1 (wave 5, 24.8%). This likely reflected a combination of factors, such as the immune vulnerability of the population and the inherent characteristics of the variants (ie, transmissibility, virulence, and immune evasiveness) [25, 28]. Following this, there was a gradual decline in positivity rate, potentially reflecting the growing immunity in the local population with each new wave [29, 30]. The Delta variant of concern has been reported to have increased odds of causing symptomatic and severe disease as compared with the ancestral, Alpha, and Omicron variants [31, 32]. The local seroprevalence stood at 24.7% (95% CI, 17.5%–32.6%) at the start of the Delta wave [33].

Notably, the interval between waves has been increasing over time from a few weeks to nearly annual from 2024 to 2025, suggesting that the SARS-CoV-2 epidemics could be settling to possibly a seasonal annual epidemic pattern, although longer surveillance is required to confirm this. With this potential seasonal circulation, targeted COVID-19 surveillance can be integrated into existing multipathogen ARI monitoring programs to improve COVID-19 management and control. In the local population, preexisting IgG antibodies that could neutralize the ancestral virus in vitro were reported in 42% of prepandemic samples [34]. However, over time, the SARS-CoV-2 seroprevalence grew to 77.4% by May 2022 in coastal Kenya, with limited neutralizing immune responses reported among emerging Omicron subvariants [29, 30]. Furthermore, ∼30% of the adult population reported receiving at least 1 dose of the COVID-19 vaccines. This suggests that local COVID-19 population immunity has primarily accrued from natural exposure to the virus [35]. We speculate that new SARS-CoV-2 wave emergence requires significant waning of previous immune responses and the arrival of a new variant with sufficient immune escape, high infectivity, and transmissibility.

Our previous work found that the observed lineages predominating different waves were introduced through international and local human mobility routes in the region. Here we confirm disparities in the local and global predominating variants in 4 waves (waves 4, 7, 8, and 10). This indicates that the SARS-CoV-2 variant that predominates globally may not necessarily predict local epidemic variant composition, as there is region-specific SARS-CoV-2 variant predominance and replacement. In turn, this emphasizes the need for sustained local genomic surveillance to inform data-driven public health responses. Furthermore, variants including FY.4.1, predominant in wave 7, have been shown to have likely emerged locally within Kenya and then spread globally such that some global waves can be driven by locally emerging variants [14].

Like studies conducted elsewhere [8, 11, 12, 36], we observed that some clinical symptoms are predictive of COVID-19 disease, and there has been a shift in SARS-CoV-2 clinical presentation from the pre-Omicron to Omicron periods. Early in the pandemic, SARS-CoV-2 infections uniquely presented with symptoms including loss of smell and taste and fatigue. The Delta variant infections largely presented with moderate to severe lower respiratory tract symptoms, causing millions of fatalities, while Omicron infections present with mild upper respiratory symptoms and systemic symptoms [8, 9, 37]. These changes could also be attributed to the growing population immunity from natural infections with more waves, as <30% of the local population received COVID-19 vaccination [29, 30, 33]. The clinical presentation of SARS-CoV-2 infections became increasingly indistinguishable from other common respiratory viruses [38].

Among Omicron subvariants (eg, BA.1, BA.2, BA.5, and XBB), differences in clinical profiles have been described as observed here [11, 36]. The emergence of the JN.1 variant led to an upsurge of cases globally, and in this study, we observed that when compared with these variants, Omicron BA.1/2 was strongly associated with joint pain, headache, body malaise, sore throat, nasal discharge, and nasal flaring; Omicron BA.4/5 was strongly associated with body malaise, sore throat, chest pain, and nasal discharge; and XBB-like variants were strongly associated with sore throat and cough. These findings show that routine linkage of syndromic surveillance with genomic data is critical in understanding the evolving clinical presentation of SARS-CoV-2 infection, thereby informing the formulation of guidelines on case definitions, clinical diagnosis, and management by clinicians.

This study had limitations. First, only a fraction of symptomatic ARI cases were enrolled in the 5 outpatient clinics (∼15 cases per week). Our study did not include asymptomatic or mildly symptomatic cases, which make the greatest fraction of COVID-19 cases in Africa [7]. Our previous work based on community surveillance indicated that up to 83% of local SARS-CoV-2 infections were asymptomatic [3]. However, similar strains were identified in infections irrespective of symptom status. Second, facility-based surveillance may be biased by the background population’s health-seeking behavior, and our study catchment area in Kenya is relatively small (∼900 km^2^). This notwithstanding, we have shown that COVID-19 epidemiology including circulating strains is comparable across regions in Kenya [23]. Third, we did not assess the SARS-CoV-2 immune status of the study participants, which may influence the COVID-19 clinical profile. Fourth, our analysis on associations between clinical symptoms and SARS-CoV-2 positivity or negativity encountered small subgroup numbers resulting in sometimes uncertain estimates. Finally, our surveillance system does identify participants on repeat visits.

In conclusion, using a sustained outpatient surveillance platform, we show the evolution of SARS-CoV-2 wave dynamics on the Kenyan Coast over the past 5 years. Within waves of infection, positivity among ARI cases has decreased, and interwave intervals has increased over the years. Distinct PANGO lineages dominated different waves, some clearly introduced from other global locations with others unique to our location. The clinical presentation has evolved from the pre-Omicron to Omicron period and can be distinct even among the Omicron subvariants. Sustained sentinel surveillance that links syndromic and genomic surveillance is crucial in identifying emerging SARS-CoV-2 variants, updating knowledge on SARS-CoV-2 clinical profiles, and predicting the nature and timing of future waves.

## Supplementary Material

ofag084_Supplementary_Data

## Supplementary Data

Supplementary materials are available at *Open Forum Infectious Diseases* online. Consisting of data provided by the authors to benefit the reader, the posted materials are not copyedited and are the sole responsibility of the authors, so questions or comments should be addressed to the corresponding author.
