# Supplementary material for: Longitudinal Epidemiology and Variant Dynamics of SARS-CoV-2 in Coastal Kenya (2020–2025): Clinical Features and Wave Patterns
Source: Open Forum Infect Dis. 2026 Feb 18;13(3):ofag084. doi: 10.1093/ofid/ofag084 (PMC12996870; doi:10.1093/ofid/ofag084)
Supplement: ofag084_Supplementary_Data [file ofag084_supplementary_data.docx]

Supplementary material

Longitudinal Epidemiology and Variant Dynamics of SARS-CoV-2 in Coastal Kenya (2020–2025): Clinical Features and Wave Patterns

Table of Contents

[Supplementary Material and Methods 2](#_Toc218621945)

[SARS-CoV-2 screening 2](#_Toc218621946)

[SARS-CoV-2 whole genome sequencing (WGS) 2](#_Toc218621947)

[Genomic data analysis 3](#_Toc218621948)

[Nanopore data 3](#_Toc218621949)

[Illumina data 3](#_Toc218621950)

[Presenting symptoms 3](#_Toc218621951)

[Data collection and management 4](#_Toc218621952)

[Supplementary tables 5](#_Toc218621953)

[Supplementary table 1: Adjusted odds ratio showing association between SARS-CoV-2 infection status and individual symptoms 5](#_Toc218621954)

[Supplementary Table 2: Grouping of SARS-CoV-2 lineages into variants 6](#_Toc218621955)

[Supplementary Table 3: Comparison of demographic characteristics and individual symptoms across different Omicron variants from health facility surveillance in Kilifi, Kenya. 8](#_Toc218621956)

[Supplementary Table 4: Adjusted odds ratio showing association between Omicron variants and individual symptoms 10](#_Toc218621957)

[Supplementary Figures 11](#_Toc218621958)

[Supplementary Figure 1: Frequency of detected SARS-CoV-2 lineages in this study. 11](#_Toc218621959)

[Supplementary Figure 2: Symptom epidemiology in SARS-CoV-2 positive individuals seeking outpatient care. 12](#_Toc218621960)

[References 13](#_Toc218621961)

# Supplementary Material and Methods

## SARS-CoV-2 screening

To screen for SARS-CoV-2, the following commercial kits/or in-house protocols were used: (1) Sansure Biotech Novel Coronavirus (2019-nCoV) Nucleic Acid Diagnostic real-time RT-PCR kit, (2) TaqPath™ COVID-19 Fast PCR Kit (N gene), (3) European Virus Archive – GLOBAL (EVA-g) primers (targeting E or RdRp or ORF1AB genes) and (4) an in-house real-time RT-PCR with primers/probe targeting the envelope (E) gene (forward: 5'- ACA GGT ACG TTA ATA GTT AAT AGC GT -3', reverse: 5'- ATA TTG CAG CAG TAC GCA CAC A -3' and probe 5'-ACA CTA GCC ATC CTT ACT GCG CTT CG-3'). For PCR Mastermix we used QIAGEN Multiplex RT-PCR + R Kit (QIAGEN, UK). Samples were assigned a positive status based on kit/protocol determined cut-offs ^1,2^. For quality control, negative and positive controls were included both at the extraction and RT-PCR stages as per kit recommendations.

## SARS-CoV-2 whole genome sequencing (WGS)

Ribonucleic acids (RNA) were re-extracted from 140µl of positive samples using the QIAamp Viral RNA Mini Kit (QIAGEN, UK). 8ul of RNA was reverse-transcribed using 2ml of LunaScript RT Mix (NEB, E3010, MA, USA) by incubating at 25◦C for 2 min, 55 °C for 10 min and 95 °C for 10 min. The cDNA was the PCR amplified using ARTIC primers V5.3.2 (https://github.com/quick-lab/SARS-CoV-2) in 2 pools by combining 1.3ul of cDNA, 2μl of primer pool, 6.3μl of Q5 Hot Start High-Fidelity 2X Master Mix (NEB M0494, MA, USA) and 1.9μl of nuclease-free water ^2,3^. The thermocycling conditions were: 1 cycle of 98°C for 30 s, followed by 25 cycles of 98 °C for 30 s and 65 °C for 5 minutes, 15 cycles of 62.5 °C for 5 minutes and 98 °C for 15 seconds and 1 cycle of 62.5 °C for 5 minutes. The two reactions were pooled, cleaned using 1X AMPure XP beads (Beckman Coulter, A63881, Indianapolis, USA). An *ad hoc* quality control strategy described previously was used to exclude samples with low concentrations (<28 ng/ml) ^3^. Valid runs included positive and negative control worked well and samples were only taken forward if adequate quantities as inferred following Qubit analysis (≥ 18ng/μl) or expected band visualization electrophoresis gels. Libraries were prepared as per the ARTIC SARS-CoV-2 sequencing protocol ^4^ and sequenced on the Oxford Nanopore Technologies (ONT) GridION or using the COVIDSeq Assay (Illumina US) and sequenced on the Illumina MiSeq platform.

## Genomic data analysis

### Nanopore data

For ONT data assembly was done using the ARTIC Bioinformatics pipeline. Here, basecalling was done from either fast5 or pod5 data on the GridION platform to generate fastq reads. Human reads were not removed. Fastq reads were aggregated per barcode and filtered based on read length (minimum: 350 and maximum: 700) using the artic guppyplex module. Reference alignment of the filtered reads to the MN908947.3 reference was done using minimap v2.17. The alignments were then sorted and indexed using samtools v1.10. using the align_trim module, primer sequences were removed by softmasking the read alignments, number of read alignments to each amplicon normalised, reads with imperfect primer pairing removed and variants called using Clair3 v0.1. The called variants were merged from different read groups, filtered through a set of workflow specific checks, normalized using bcftools v1.10.2 and a consensus called with a minimum read depth of 20 reads per read group otherwise a position is assigned ambiguous nucleotide “N”.

### Illumina data

For samples sequenced on the Illumina Plattform, genome assembly was done using an Inhouse bioinformatics pipeline. Here, the fastq reads from the Illumina Miseq platform were quality-filtered using fastp v.0.23.4 (Phred score ≥ 30 and adapters removed). The trimmed fastq reads were mapped to the Wuhan reference (MN908947.3) using bwa v0.7.1, sorted and indexed using samtools v1.10. Primer sequences were removed and the consensus generated with a minimum depth of 10 reads using iVar v1.4.2.

## Presenting symptoms

After providing consent, the following clinical symptoms were recorded for each recruited participant as present or absent: body malaise, sore throat, headache, chest pain, fever, cough, nasal discharge, loss of sense of smell, lack of appetite, difficulty in breathing, nasal flaring, crackles, abdominal pains, dizziness, vomiting, wheezes, diarrhoea, back pain, joint pain, sneezing, epigastric pain, nausea, indrawing, body ache and feed unable.

## Data collection and management

Demographic data (e.g., date of birth, sex, location/facility), COVID‑19 vaccination status, and presenting symptoms were captured using a paper questionnaire that was jointly completed by a field assistant and attending clinicians after interviewing the participant or guardian. These data were later transferred to Redcap v.14.3.3 (<https://project-redcap.org/>). All subsequent data processing and statistical analyses were conducted in using scripts written in either Python v3.13 or R v4.2.

# Supplementary tables

## Supplementary table 1: Adjusted odds ratio showing association between SARS-CoV-2 infection status and individual symptoms

| **Symptoms** | **Entire period (2020-2025) (n=14,547)** | | **Pre-Omicron (n=2,248)** | | **Omicron (n=12,299)** | |
| --- | --- | --- | --- | --- | --- | --- |
|  | **aOR (95% C.I)** | **p value** | **aOR (95% C.I)** | **p value** | **aOR (95% C.I)** | **p value** |
| abdominal pains | 0.847 (0.522, 1.303) | 0.476 | 0.746 (0.341, 1.449) | 0.423 | 0.935 (0.492, 1.612) | 0.824 |
| back pain | 1.145 (0.465, 2.426) | 0.744 | 0.65 (0.151, 1.935) | 0.493 | 2.046 (0.597, 5.35) | 0.189 |
| body ache | 1.112 (0.173, 4.042) | 0.890 | 0.731 (0.039, 4.096) | 0.770 | – | – |
| body malaise | 1.352 (1.175, 1.552) | 0.00000212 | 1.175 (0.897, 1.53) | 0.237 | 1.424 (1.208, 1.674) | 0.0000213 |
| chest pain | 1.111 (0.87, 1.402) | 0.383 | 0.966 (0.623, 1.453) | 0.873 | 1.188 (0.881, 1.571) | 0.243 |
| cough | 0.805 (0.639, 1.025) | 0.071 | 0.643 (0.467, 0.897) | 0.008 | 1.042 (0.738, 1.523) | 0.822 |
| crackles | 0.665 (0.443, 0.959) | 0.038 | 0.277 (0.083, 0.684) | 0.014 | 0.843 (0.544, 1.247) | 0.416 |
| diarrhea | 1.196 (0.579, 2.208) | 0.596 | 0.934 (0.274, 2.407) | 0.899 | 1.517 (0.586, 3.231) | 0.330 |
| difficulty in breathing | 0.675 (0.525, 0.855) | 0.002 | 0.755 (0.511, 1.086) | 0.142 | 0.636 (0.453, 0.868) | 0.006 |
| dizziness | 1.186 (0.644, 2.018) | 0.556 | 0.643 (0.189, 1.647) | 0.411 | 1.724 (0.835, 3.18) | 0.106 |
| epigastric pain | 0.389 (0.151, 0.816) | 0.025 | 0.383 (0.09, 1.107) | 0.120 | 0.359 (0.088, 0.957) | 0.081 |
| feed unable | 0.411 (0.023, 1.962) | 0.385 | 0.734 (0.04, 3.834) | 0.768 | – | – |
| fever | 0.852 (0.75, 0.969) | 0.015 | 0.819 (0.641, 1.046) | 0.110 | 0.874 (0.751, 1.017) | 0.080 |
| headache | 1.582 (1.331, 1.871) | 0.000000129 | 1.454 (1.089, 1.926) | 0.010 | 1.658 (1.336, 2.041) | 0.00000284 |
| indrawing | 0.587 (0.178, 1.428) | 0.303 | 0.333 (0.018, 1.649) | 0.288 | 0.761 (0.185, 2.058) | 0.644 |
| joint pain | 1.166 (1.014, 1.338) | 0.0302 | 1.138 (0.877, 1.47) | 0.326 | 1.183 (1.002, 1.393) | 0.045 |
| lack of appetite | 2.146 (0.857, 4.668) | 0.073 | 1.941 (0.541, 5.545) | 0.251 | 2.597 (0.61, 7.622) | 0.125 |
| loss of smell | 6.114 (2.417, 14.967) | 0.0000796 | 10.466 (3.228, 39.82) | 0.000161 | 1.89 (0.102, 10.393) | 0.550 |
| nasal discharge | 1.338 (1.153, 1.557) | 0.000144 | 1.329 (0.99, 1.808) | 0.064 | 1.342 (1.131, 1.599) | 0.000887 |
| nasal flaring | 0.828 (0.578, 1.151) | 0.280 | 0.48 (0.261, 0.813) | 0.014 | 1.324 (0.845, 1.98) | 0.194 |
| nausea | 1.492 (0.507, 3.526) | 0.409 | 1.378 (0.207, 5.468) | 0.686 | 1.631 (0.388, 4.639) | 0.423 |
| sneezing | 1.311 (0.448, 3.071) | 0.573 | – | – | 2.858 (0.966, 6.835) | 0.032 |
| sore throat | 1.137 (0.986, 1.309) | 0.076 | 0.984 (0.764, 1.262) | 0.897 | 1.23 (1.034, 1.457) | 0.018 |
| vomiting | 0.705 (0.37, 1.219) | 0.246 | 0.61 (0.211, 1.401) | 0.296 | 0.818 (0.345, 1.627) | 0.605 |
| wheezes | 0.467 (0.238, 0.82) | 0.015 | 0.342 (0.103, 0.839) | 0.039 | 0.587 (0.249, 1.164) | 0.170 |

## Supplementary Table 2: Grouping of SARS-CoV-2 lineages into variants

| Variant |  |  | PANGO Lineages |
| --- | --- | --- | --- |
| Ancestral | – | _ | B.1.530 & A.23.1 |
| Alpha | – | Alpha (B.1.1.7) | B.1.1.7 |
| Beta | – | Beta (B.1.351) | B.1.351 |
| Delta | – | Delta (B.1.617.2/AY*) | AY.116, AY.122, AY.16, AY.46, AY.46.5 & B.1.617.2 |
| Omicron | Omicron BA.1/2 | Omicron (BA.1*) | BA.1, BA.1.1, BA.1.1.1, BA.1.1.4, BA.1.18 & BC.2 |
|  |  | Omicron (BA.2*) | BA.2 & BA.2.31.1, |
|  | Omicron BA.4/5 | Omicron (BA.4*) | BA.4, BA.4.1 & BA.4.6, |
|  |  | Omicron (BA.5*) | BA.5, BA.5.2 & BA.5.2.1 |
|  |  | Omicron (BQ*) | BQ.1, BQ.1.1, BQ.1.1.51, BQ.1.23, BQ.1.8 & BE.1 |
|  |  | Omicron (BF*) | BF.20, BF.35 & BF.9, |
|  | Omicron BA.2.86.1 | Omicron (JN.1*) | JN.1, JN.1.1, JN.1.16.1, JN.1.18, JN.1.4, JN.1.4.7, LE.1, LE.1.1 & LE.1.3 |
|  |  | Omicron (LF.1) | LF.1 |
|  |  | Omicron (LF.7*) | LF.7, LF.7.1.2, LF.7.3, LF.7.3.2 & LF.7.9, |
|  |  | Omicron (KP*) | KP.2.3, KP.3.1.1 LP.8.1, MV.1 & PC.1 |
|  |  | Omicron (MV.1*) | MV.1 |
|  |  | Omicron (LP.8) | LP.8.1 |
| Recombinant | Recombinant | Recombinant (FY.4*) | FY.4.1, FY.4.1.1 & FY.4.1.2 |
|  |  | Recombinant (XBB.2.3*) | GE.1.2, GE.1.2.1, GE.1.2.2, GS.4.1, KH.1, KT.1 & KT.1.2, |
|  |  | Recombinant (XEF) | XEF |
|  |  | Recombinant (XBB.1*) | XBB.1 & XBB.1.3.1, |
|  |  | Recombinant (XBB.3 | XBB.3 |
| VOI | – | Eta (B.1.525) | B.1.525 |

## Supplementary Table 3: Comparison of demographic characteristics and individual symptoms across different Omicron variants from health facility surveillance in Kilifi, Kenya.

|  | Omicron BA.1/2 (n=84) | Omicron BA.4/5 (n=224) | XBB (n=132) | Omicron BA.2.86.1 (n=138) | Total (n=578) | *p value* |
| --- | --- | --- | --- | --- | --- | --- |
| Sex |  |  |  |  |  | **0.185** |
| Female | 52 (61.9%) | 166 (74.1%) | 89 (67.4%) | 95 (68.8%) | 402 (69.6%) |  |
| Age Group |  |  |  |  |  | **< 0.001** |
| 0 - 4 | 5 (6.0%) | 13 (5.8%) | 29 (22.0%) | 12 (8.7%) | 59 (10.2%) |  |
| 5 - 9 | 11 (13.1%) | 11 (4.9%) | 9 (6.8%) | 12 (8.7%) | 43 (7.4%) |  |
| 10 - 19 | 9 (10.7%) | 62 (27.7%) | 21 (15.9%) | 23 (16.7%) | 115 (19.9%) |  |
| 20 - 39 | 35 (41.7%) | 82 (36.6%) | 37 (28.0%) | 51 (37.0%) | 205 (35.5%) |  |
| 40 - 64 | 23 (27.4%) | 48 (21.4%) | 24 (18.2%) | 31 (22.5%) | 126 (21.8%) |  |
| 65+ | 1 (1.2%) | 8 (3.6%) | 12 (9.1%) | 9 (6.5%) | 30 (5.2%) |  |
| Clinical presentation |  |  |  |  |  |  |
| Body malaise | 36 (42.9%) | 82 (36.6%) | 36 (27.3%) | 37 (26.8%) | 199 (33.0%) | **0.025** |
| Sore throat | 36 (42.9%) | 63 (28.1%) | 37 (28.0%) | 23 (16.7%) | 159 (27.5%) | **< 0.001** |
| Headache | 25 (29.8%) | 47 (21.0%) | 14 (10.6%) | 18 (13.0%) | 104 (18.0%) | **< 0.001** |
| Chest pain | 6 (7.1%) | 33 (14.7%) | 1 (0.8%) | 3 (2.2%) | 43 (7.4%) | **< 0.001** |
| Fever | 43 (51.2%) | 112 (50.0%) | 96 (72.7%) | 88 (63.8%) | 339 (58.7%) | **< 0.001** |
| Cough | 82 (97.6%) | 210 (93.8%) | 131 (99.2%) | 129 (93.5%) | 552 (95.5%) | 0.044 |
| Nasal discharge | 69 (82.1%) | 177 (79.0%) | 98 (74.2%) | 92 (66.7%) | 436 (75.4%) | **0.024** |
| Lack of appetite | 0 (0.0%) | 1 (0.4%) | 0 (0.0%) | 0 (0.0%) | 1 (0.2%) | 0.663 |
| Difficulty breathing | 4 (4.8%) | 11 (4.9%) | 7 (5.3%) | 4 (2.9%) | 26 (4.5%) | 0.771 |
| Nasal flaring | 6 (7.1%) | 3 (1.3%) | 4 (3.0%) | 2 (1.4%) | 15 (2.6%) | **0.028** |
| Crackles | 4 (4.8%) | 11 (4.9%) | 4 (3.0%) | 0 (0.0%) | 19 (3.3%) | 0.067 |
| Abdominal pains | 2 (2.4%) | 2 (0.9%) | 0 (0.0%) | 3 (2.2%) | 7 (1.2%) | 0.279 |
| Dizziness | 1 (1.2%) | 7 (3.1%) | 0 (0.0%) | 0 (0.0%) | 8 (1.4%) | **0.032** |
| Vomiting | 1 (1.2%) | 2 (0.9%) | 0 (0.0%) | 1 (0.7%) | 4 (0.7%) | 0.716 |
| Wheezes | 0 (0.0%) | 3 (1.3%) | 1 (0.8%) | 0 (0.0%) | 4 (0.7%) | 0.404 |
| Diarrhea | 1 (1.2%) | 0 (0.0%) | 0 (0.0%) | 1 (0.7%) | 2 (0.3%) | 0.315 |
| Backpain | 0 (0.0%) | 3 (1.3%) | 0 (0.0%) | 0 (0.0%) | 3 (0.5%) | 0.19 |
| Joint pains | 39 (46.4%) | 71 (31.7%) | 36 (27.3%) | 33 (23.9%) | 179 (31.0%) | 0.004 |
| Sneezing | 1 (1.2%) | 1 (0.4%) | 2 (1.5%) | 0 (0.0%) | 4 (0.7%) | 0.43 |
| Epigastric pain | 2 (2.4%) | 1 (0.4%) | 0 (0.0%) | 0 (0.0%) | 3 (0.5%) | 0.07 |
| Nausea | 0 (0.0%) | 2 (0.9%) | 0 (0.0%) | 0 (0.0%) | 2 (0.3%) | 0.366 |
| Indrawing | 0 (0.0%) | 2 (0.9%) | 0 (0.0%) | 0 (0.0%) | 2 (0.3%) | 0.366 |
| Body ache | 0 (0.0%) | 1 (0.4%) | 0 (0.0%) | 0 (0.0%) | 1 (0.2%) | 0.663 |

## Supplementary Table 4: Adjusted odds ratio showing association between Omicron variants and individual symptoms

| **Variant** | **n** | **Symptoms** | **Adjusted odds ratio (95% C.I)** | **p value** |
| --- | --- | --- | --- | --- |
| Omicron-BA.1/2 | 84 | Joint pains | 2.83 (1.58, 5.12) | <0.001 |
|  |  | headache | 2.84 (1.44, 5.71) | 0.002 |
|  |  | body malaise | 2.07 (1.16, 3.71) | 0.01 |
|  |  | sore throat | 3.94 (2.11, 7.48) | <0.001 |
|  |  | Nasal discharge | 2.29 (1.20, 4.56) | 0.014 |
|  |  | nasal flaring | 5.26 (1.17, 36.62) | <0.001 |
| Omicron BA.4/5 | 224 | Body malaise | 1.60 (1.01, 2.57) | <0.001 |
|  |  | Sore throat | 1.99 (1.17, 3.47) | 0.01 |
|  |  | Chest pain | 8.03 (2.79, 33.97) | <0.001 |
|  |  | Nasal discharge | 1.86 (1.15, 3.02) | 0.01 |
| XBB-like | 132 | Sore throat | 2.05 (1.14, 3.75) | 0.017 |
|  |  | Cough | 8.95 (1.64, 166.39) | 0.039 |
| *Reference group: Omicron BA.2.86.1* | | | | |

# Supplementary Figures

Supplementary Figure 1: Frequency of detected SARS-CoV-2 lineages in this study. The lineages were detected between December 2020 and February 2025 in SARS-CoV-2 cases presenting with ARI symptoms in five outpatient healthcare facilities.

Supplementary Figure 2: Symptom epidemiology in SARS-CoV-2 positive individuals seeking outpatient care. (A) Prevalence of COVID-19 symptoms in the pre-Omicron and Omicron periods. Error bars indicate 95% credible intervals (Cis). (B) Frequency of symptoms reported per individual. The dotted line shows the median number of symptoms reported per individual. (C) Prevalence of COVID-19 symptoms by age. A cut-off of five years and above was used to avoid bias among the symptoms as some cannot be recorded for children (e.g., headache). A gradient colour scale is used to show high and low proportions.

# References

1. Mohammed KS, de Laurent ZR, Omuoyo DO, Lewa C, Gicheru E, Cheruiyot R, et al. An optimization of four SARS-CoV-2 qRT-PCR assays in a Kenyan laboratory to support the national COVID-19 rapid response teams. Wellcome Open Res [Internet]. 2022 Mar 4;5:162. Available from: https://wellcomeopenresearch.org/articles/5-162/v2

2. Lambisia AW, Katama EN, Moraa E, Mwita JM, Gallagher K, Mutunga M, et al. Genomic and clinical epidemiology of SARS-CoV-2 in coastal Kenya: Insights into variant circulation, reinfection, and multiple lineage importations during a post-pandemic wave. medRxiv [Internet]. 2025; Available from: https://www.medrxiv.org/content/early/2025/03/27/2025.03.26.25324476

3. Lambisia AW, Mohammed KS, Makori TO, Ndwiga L, Mburu MW, Morobe JM, et al. Optimization of the SARS-CoV-2 ARTIC Network V4 Primers and Whole Genome Sequencing Protocol. Front Med (Lausanne) [Internet]. 2022 Feb 17;9(February):1–8. Available from: https://www.frontiersin.org/articles/10.3389/fmed.2022.836728/full

4. Quick J, Lansdowne L. ARTIC SARS-CoV-2 sequencing protocol v4 (LSK114) v2. 2024.
